# Comparative Study of Antimicrobial Properties of Bee Venom Extracts and Melittins of Honey Bees

**DOI:** 10.3390/antibiotics10121503

**Published:** 2021-12-08

**Authors:** Jakkrawut Maitip, Wannapha Mookhploy, Supharerk Khorndork, Panuwan Chantawannakul

**Affiliations:** 1 Faculty of Science, Energy and Environment, King Mongkut’s University of Technology North Bangkok, Rayong Campus, Rayong 21120, Thailand; jakkrawut.m@sciee.kmutnb.ac.th; 2 Bee Protection Laboratory, Department of Biology, Faculty of Science, Chiang Mai University, Chiang Mai 50200, Thailand; wannapha.mookhploy@gmail.com (W.M.); k.supharerk1@gmail.com (S.K.); 3 Environmental Science Research Center (ESRC), Faculty of Science, Chiang Mai University, Chiang Mai 50200, Thailand

**Keywords:** bee venom, eastern honey bee, apitherapy

## Abstract

Bee venom (BV), or apitoxin, is a complex substance produced by a gland in the abdominal cavity of bees. The main component of BV is melittin, which is a largely studied substance due to its biological properties. To date, the most well-known bee venom and melittin are derived from domesticated honey bees, while venom and melittin derived from wild honey bees have been under-investigated. Hence, this study primarily reports the antimicrobial activities of bee venom and synthetic melittin derived from four different honey bee species (*Apis mellifera*, *A. cerana, A. dorsata*, and *A. florea*) in Thailand. All the bee venom extracts and melittins showed more robust antibacterial activities against Gram-positive (*Bacillus subtilis*, *Micrococcus luteus*, *Staphylococcus aureus*, *S. aureus MRSA*, and *S. epidermidis*) than Gram-negative bacteria (*Escherichia coli*, *Klebsiella pneuminiae*, and *Salmonella typhimurium*) or a fungus (*Candida albicans*), while the synthetic melittins also have antimicrobial activity at higher concentrations than the bee venom extract. Furthermore, the *A. cerana* venom extract showed the highest activity against the tested bacteria, followed by *A. mellifera*, *A. florea,* and *A. dorsata*. Therefore, *A. cerana* venom may be further developed for use in medical applications as a potential alternative agent against Gram-positive bacteria and antibiotic-resistant bacteria.

## 1. Introduction

The honey bee is an essential economic insect; it is widely used in agriculture as a pollinator and produces several natural products. These products, such as honey, pollen, royal jelly, propolis, and, especially, honey bee venom, or Apitoxin, can be applied in the fields of pharmacy and medicine. Bee venom is produced by the venom glands located in the posterior of the abdomen and is stored in the venom reservoir. The venom is released through the sting apparatus to protect the bee from harm [1]. The Western honey bee’s (*Apis mellifera*) venom has been used since the ancient Egyptian era (4000 BC) [2]. Traditional medicine has been utilized to treat several diseases, including arthritis, rheumatism, pain, cancerous tumors, and skin diseases [3]. Extensive research has been carried out regarding the Western honey bee; it was found that its venom has multiple effects, consisting of anti-inflammatory [4], antimicrobial [2,5,6,7], and antioxidant activities [4,8].

Honey bee venom contains a variety of active substances, including peptides (e.g., melittin, apamin, adolapin, and mast cell degranulating peptide); enzymes (e.g., phospholipase A2 (PLA_2_) and hyaluronidase); biologically active amines (e.g., histamine and epinephrine); and non-peptide components (including lipids, carbohydrates, and free amino acids) [2]. The major component of bee venom is melittin, which accounts for 40–50% of the venom’s dry weight [3]. Melittin is a cationic peptide and an amphipathic peptide of 26 amino acid residues; residues 1–20 are hydrophobic in the amino-terminal end [9], and residues 21–26 are hydrophilic in the carboxy-terminal end. It can dissolve in water as a monomer or tetramer [10] and is a principal active component of bee venom that displays high antimicrobial and cytotoxic properties [11].

To our knowledge, the antimicrobial activities of different bee venoms and melittins have not yet been investigated. The venom of *Apis mellifera* has been widely studied in Hymenoptera, whereas there is limited information regarding the native honey bee’s venom. Few studies have reported on the antioxidant activity of the crude venom of *A. dorsata* [8] and anticancer activity of the melittin of *A. florea*.

Interestingly, differences between different honey bee species affect the compositions of each of their venoms. The melittin contents vary in different honey bees; for example, the levels in *A. dorsata*, *A. mellifera*, *A. florea*, and *A. cerana* were 95.8 ± 3.2%, 76.5 ± 1.9%, 66.3 ± 8.6%, and 56.8 ± 1.8%, respectively [8]. The size of the venom gland and venom sac secretion depends on the body size of the honey bee, which means *A. dorsata* possesses the largest venom gland and venom sac, followed by *A. cerana*, *A. mellifera*, and *A. florea*. Moreover, the amount and composition of the lipid, protein, carbohydrate, and alkaline phosphatase are usually the highest in *A. cerana*, followed by *A. mellifera* and *A. florea*. The antimicrobial activity of bee venom and melittin from different honey bee species is poorly understood. This study collected crude venom from four honey bee species, and the melittin genes were discovered and used as the template for melittin synthesis. The antimicrobial activity of crude venom and synthetic melittin were compared against bacteria and a fungus.

Our findings showed that the crude venom and melittin taken from *A. cerana* are the most effective in antimicrobial activity against Gram-positive bacteria and *MRSA* as opposed to Gram-negative bacteria and the fungus, followed by *A. mellifera*, *A. florea*, and *A. dorsata*. The crude bee venom showed higher antimicrobial activity compared to synthetic melittin. Based on these results, we suggest that different honey bee species have different melittin amino acid compositions that affect the antimicrobial activity of their venom, and crude bee venom is more effective with regard to antimicrobial activity than melittin alone.

## 2. Results

### 2.1. Melittin Sequence

The total genomic DNA was extracted from native honey bees (*A. florea* and *A. dorsata*). The melittin gene sequences were obtained after PCR amplification of their genomic DNA deposited in GenBank (Table 1). To synthesize four types of melittin peptides, we used two melittin gene sequences derived in this study and the other two melittin gene sequences retrieved from the NCBI database (*A. mellifera* and *A. cerana*) as a template for melittin peptides synthesis (Table 1).

**Figure 1:**
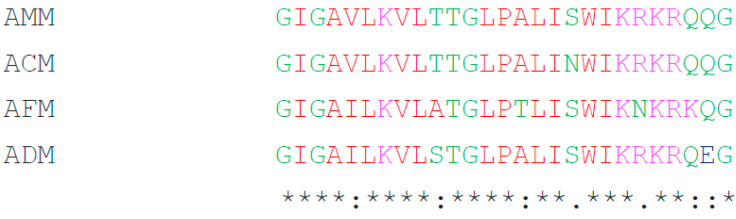
Multiple sequence alignment of melittin peptides from four honey bee species via MUSCLE. An asterisk (*) indicates highly conserved sites, and a colon (:) and dot (.) represent less-conserved sites.

The multiple sequence alignments of four melittin amino acid sequences were predicted using MUSCLE (Figure 1). The results showed that the melittin of *A. mellifera* (AMM) was similar to *A. cerana* (ACM) (96.30%) but different from *A. dorsata* (ADM) (85.19%) and *A. florea* (AFM) (77.78%).

### 2.2. Amino Acid Composition

The amino acid composition of the melittin sequences was computed using ExPAsy ProtParam. The primary sequence analysis results are shown in Table 2. The results from the ExPAsy server (Table 2) indicated that the melittin from the different honey bees had 27 amino acids, and the protein had an average molecular weight that ranged from 2876.52 to 2931.56 Da. The similarity of the amino acid composition ranged from 77.78% to 96.3%. The analysis revealed that the most abundant amino acid residues in melittin are glycine, leucine, isoleucine, and lysine. Based on the instability index (II), ExPAsy ProtParam classified the AMM and ADM as unstable (II > 40) and classified the ACM and AFM as stable (II < 40). The computed pI values of all the melittin samples were higher than 7, which indicated that these melittins were basic in nature. The extinction coefficient (~5500) indicated that the quantity of light that the protein could absorb was computed using tyrosine, tryptophan, and cysteine, while the aliphatic index ranged from 130 to 133.7, GRAVY ranged from 0.148 to 0.281, and the instability index ranged from 34.64 to 50.58.

### 2.3. Secondary Structure

**Figure 2:**
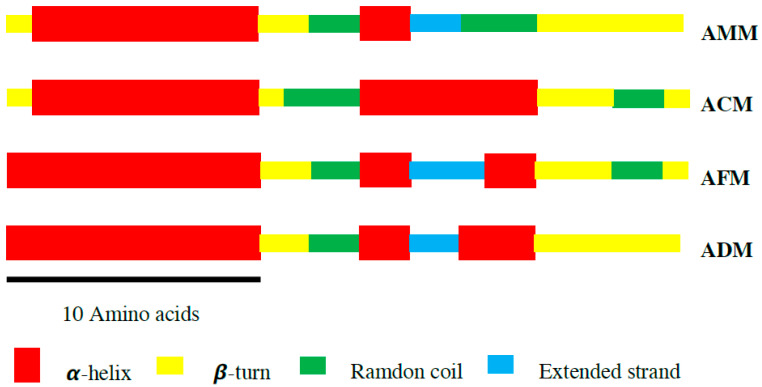
Schematic representation of the predicted molecular architecture of melittin from four honey bee species based on the secondary structural analysis via SOPMA. The computation of melittin from different honey bees showed a predominance of α-helices (red), followed by β-turns (yellow), random coils (green), and extended strands (blue). The scale bar represents the lengths of 10 amino acids.

SOPMA computed the secondary structure of the melittin samples. The secondary structure of the melittin from four *Apis* species was predicted to contain an a-helix, b-turn, random coil, and extended strand, which revealed that the main structure of melittin is an a-helix (Figure 2). The a-helix structure in the four melittins analyzed ranged from 40.74% to 59.26%, while the extended strand was least commonly found in melittin, ranging from 7.41% to 11.11%, and not found in ACM (Table 3). The most common amino acid residues in melittin’s a-helix regions were found to be glycine, leucine, isoleucine, and lysine. The significant difference between the amino acid composition in the different melittin samples was in the 18th position in the sequence. Melittin from *A. mellifera*, *A. dorsata*, and *A. florea* was found to contain asparagine (Asn), and melittin from *A. cerana* was found to contain serine (Ser). The change in the amino acid composition in the 18th position from asparagine to serine also changed the secondary structure, particularly in the a-helix region (Figure 2).

### 2.4. Primary Sequence Analysis

**Figure 3:**
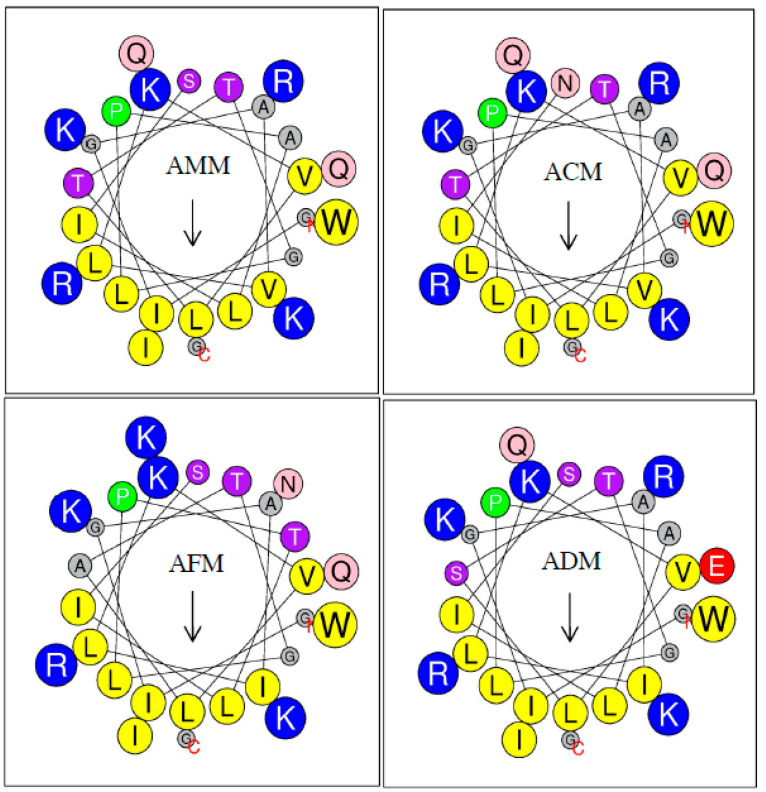
Helical wheel plots of the melittin from four honey bee species. The length of the arrow (↓) indicates the level of hydrophobicity <H> of the peptide. The size of the letter represents the size of the amino acid side chain. The hydrophilic and hydrophobic portions are labeled in blue and yellow circles, respectively.

The helical wheel plots of melittins from four *Apis* species predicted by HeliQuest [13] are shown in Figure 3. The length of the arrow indicates the extent of hydrophobicity. The color of the amino acid represents the basic residue (blue) and nonpolar residue (yellow), while the size of the circle represents the amino acid side chain. The hydrophobicity <H> and hydrophobic moment <μH> values ranged from 0.471 to 0.500 and 0.380 to 0.412, respectively.

### 2.5. Physicochemical Parameters

In this experiment, four melittin peptides were studied, including their physicochemical parameters and structures. The results shown in Table 2 reveal that melittin’s isoelectric point (pI) ranged from 11.10 to 12.02. ACM showed a high molecular weight of 2931.56 Da, while that of AFM presented a low molecular weight of 2876.52 Da. The molecular weights of ADM and AMM were approximately equal. The hydrophobicity of AMM was similar to that of AFM, but ACM possessed the lowest hydrophobicity. Only the melittin ADM presented four positive net charges, while the remaining three melittins showed five positive net charges. The four melittins’ grand average hydropathy (GRAVY) values were slightly positive, ranging from 0.148 to 0.281. The melittins’ aliphatic index (AI) was 130 for AMM and ACM and 133.7 for AFM and ADM. The melittins’ instability index (II) was divided into two groups: II < 40 for ACM and AFM and II > 40 for AMM and ADM.

### 2.6. Antimicrobial Activities of Four Crude Honey Bee Venoms

The antimicrobial activity of crude venom from four honey bee species (*A. mellifera, A. cerana, A. dorsata,* and *A. florea*) against nine microorganisms were investigated using a two-fold serial dilution assay. The results showed that the MIC and MBC values of all the crude venoms against the Gram-positive bacteria were lower than those against the Gram-negative bacteria and yeast (Table 4). *S. epidermidis* showed MIC and MBC values at the lowest concentrations out of all the crude venom types. The crude venoms of *A. cerana* and *A. mellifera* did not display statistically significant differences with regard to the MIC and MBC values (*p* = 0.395 and 0.066, respectively). Moreover, the crude venoms of *A. cerana* and *A. mellifera* possessed higher MIC and MBC values than the crude venoms of *A. florea* and *A. dorsata* (MIC and MBC, *p* < 0.05). The crude venom of *A. dorsata* displayed low activity compared with the crude venom of *A. florea* (MIC, *p* = 0.013 and MBC, *p* = 0.028).

### 2.7. Antimicrobial Activities of Four Melittin Peptides

In this experiment, four melittin peptides were synthesized to be tested with nine microorganisms. The MIC and MBC/MFC values of all the melittin peptides against the Gram-positive bacteria were lower in concentration compared to the values with regard to the Gram-negative bacteria and yeast, which are shown in Table 5. It was noticed that the MIC and MBC values of all the melittin peptides against *B. subtilis* showed high concentrations at ≥400 µg/mL, while *S. epidermis* was the most susceptible to ACM, with both the MIC and MBC values at 12.5 ± 0.0 µg/mL. All the melittin peptides were compared with regard to their potential antimicrobial activity, which showed significant differences at *p* <0.001. The MIC value of ACM showed the highest activity against the tested microorganisms compared to that of AMM, AFM, and ADM at *p* < 0.05; a similar trend was found regarding the MBC/MFC values. The comparison between AMM and AFC did not produce statistically significant different results in regard to the MIC and MBC/MFC values (*p* = 0.154 and 0.146, respectively). While the MIC and MBC/MFC values of AMM showed statistically significant differences with ADM (*p* = 0.011), the MIC value of AFC was not statistically significant with ADM (*p* = 0.107).

### 2.8. Comparison of Antimicrobial Activity between Venoms and Their Melittin Peptides

**Figure 4:**
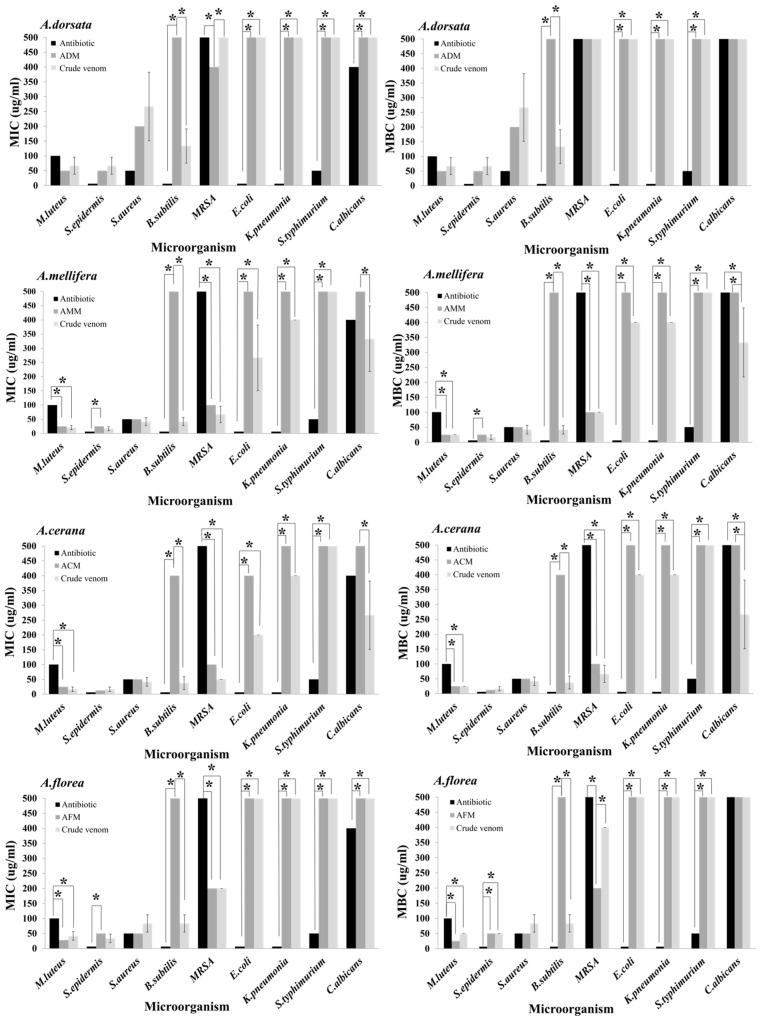
Comparison of the MIC and MBC/MFC (mean ± SD) among the crude venom, melittin peptide, and antibiotics (positive controls) in each honey bee species against the pathogenic bacteria. Asterisks (*) represent statistically significant differences at *p* < 0.05.

Gentamycin showed a lower concentration of MIC and MBC/MFC against almost all the bacteria than all the crude venoms or their melittin peptides. For example, *M. luteus* and *MRSA* were inhibited by all the crude venoms or their melittin peptides at lower concentrations than gentamycin. Incredibly, the crude venom of *A. mellifera* and *A. cerana*, as well as the melittin peptide of *A. florea*, showed statistically significant differences in terms of the MIC and MBC/MFC when compared with gentamycin at *p* < 0.05 (Figure 4). Comparisons between the crude venoms and their melittin peptides in all honey bee species against microorganisms did not reveal any statistically significant different results, except *A. dorsata* against *MRSA* (melittin produced a lower MIC value than the crude venom, *p* = 0.043), *A. mellifera*, and *A. cerana* against *C. albicans* (crude venom produced lower MIC and MFC values than the melittin peptide; MIC, *p* = 0.037 and MFC, *p* = 0.048) (Figure 4). While the crude venom of all honey bee species seemed to strongly inhibit *B. subtilis* rather than their melittin, this finding was not statistically significantly different.

## 3. Discussion

The sequences of the melittin of four honey bee species were revealed via the PCR technique, and it was found that the melittin of *A. mellifera* and *A*. *cerana* shared high similarities but displayed some differences with *A. dorsata* and *A. florea*. This investigation suggests that the genetic differences in melittin are due to the evolution of honey bee species. *A. mellifera* and *A. cerana* possess a close relationship via their genetics, and they are a multiple-comb cavity nesting species, while *A. dorsata* and *A. florea* are single-comb open-air nesting species [14]. Honey bee venom has been previously reported to kill both Gram-positive and Gram-negative bacteria. El-Seedi et al. [2] suggested that Gram-positive bacteria are more sensitive to melittin than Gram-negative bacteria due to the nature of the cell membrane layer. Melittin peptides can enter Gram-positive bacteria through the peptidoglycan layer of the Gram-positive cell membrane with less effort than in Gram-negative cells, which have a layer of lipopolysaccharides over the peptidoglycan layer to protect their membrane. In addition, the proline residue in position 14 of the melittin also plays a crucial role in the antimicrobial activity of melittin. Han et al. [5] reported that Korean *A. mellifera* showed high antimicrobial activity against Gram-positive mastitis pathogens, especially *S. aureus* and *MRSA*, as it was shown to inhibit Gram-negative bacteria (*E. coli*). Moreover, we also found that extracted venom from *A. cerana* showed the highest antimicrobial activity, followed by *A. mellifera*, *A. florea*, and *A. dorsata*. This result was in agreement with the findings of Surendra et al. [15], who measured the antimicrobial activity of venom in *Apis* using a disk diffusion assay, and *A. cerana* venom showed a higher inhibitory zone. Honey bee venom contains several bioactive substances, and the principal component is melittin, which many studies have reported as having high antimicrobial activity [16,17,18]. The negative charge of the surface of the lipid membrane of microorganisms is attracted to the positive charge of melittin. It can be inserted into the lipid bilayer and can lead to pore formation in the membrane [19]. Additionally, 7-dehydrocholesterol, stigmasterol, cholesterol, and ergosterol can decrease a membrane’s sensitivity to melittin [20]. Therefore, yeast is not sensitive to melittin, because the yeast plasma membrane contains large amounts of ergosterol [21]. Most Gram-positive bacteria are more sensitive to venom than Gram-negative bacteria because of the differences in their cell membrane structures. A single plasma membrane binds Gram-positive bacteria, whereas Gram-negative bacteria are bound by two membranous structures (the inner and outer membrane), showing different compositions [22,23].

In this experiment, the antimicrobial activities of crude venom and each melittin peptide (ADM, AMM, and AFM) were determined against standard human pathogenic microorganisms, including Gram-positive (*Bacillus subtilis*, *Micrococcus luteus*, *Staphylococcus aureus*, *S. aureus MRSA*, and *S. epidermidis*) and Gram-negative bacteria (*Escherichia coli*, *Klebsiella pneuminiae*, and *Salmonella typhimurium*) or a fungus (*Candida albicans*). Single-nucleotide polymorphisms (SNP) have been studied in the melittin gene of *A. mellifera* and *A. cerana*, and it was found that the novel SNP of the melittin gene exists in two honey bee species [12]. Hence, a novel melittin peptide (ACM) was tested for antimicrobial activity in our experiment. The results showed that microorganisms were the most susceptible to ACM, followed by AMM, AFC, and ADM. The critical factors for antimicrobial peptide efficiency were revealed, including sequence, size, structuring, charge, amphipathicity, and hydrophobicity [24]. The four positive net charges of ADM had lower antimicrobial activity than the remaining three peptides with five positive net charges. Previous research found that decreasing the protein net charge to +4 significantly reduced the antimicrobial activity of V13K for both Gram-positive and Gram-negative bacteria [25].

Furthermore, increasing the charge resulted in stronger peptide binding to the negatively charged membranes [26], implying that a higher net charge leads to an increase in antimicrobial activity. The hydrophobicity of AFM was similar to AMM, but the secondary structure of AMM had a higher α-helical potency and lower β-sheet in the partial structure than AFM. The effect of the peptide secondary structure on the antimicrobial activity has been previously studied by designing an α-helical peptide with a higher helical propensity than the original peptide, a β-sheet peptide, and a random coiled peptide. The α-helical conformation peptide showed higher antimicrobial activity, but the β-sheet and random coiled peptide lost their efficiency [27]. AMM and ACM displayed a difference in one amino acid residue at the 18th position in the melittin peptide; in AMM, this was serine (S), and in ACM, this was asparagine (N). AMM exhibited higher hydrophobicity than ACM. In 2014, Park et al. [12] found that Mel-(S) showed more antimicrobial activity than Mel-(N) at 0.25 µM, but some *E. coli* was still alive in this concentration.

In contrast, we found that ACM showed higher antimicrobial activity than AMM and ultimately killed the bacteria in some bacteria species. This result may be explained by the fact that an increase in the hydrophobicity increased the membrane disruption [28,29,30] but only if a high concentration of peptide was the cause of self-aggregation [10]. The self-aggregation in the peptide decreased the antimicrobial activity [31].

By comparing types of venom and their melittin peptides, we found that the melittin of both *A. florea* and *A. dorsata* showed stronger activity than their extracted venoms. The significant potency may be derived from melittin in their extracted venom. On the other hand, the antimicrobial effects of the *A. mellifera* and *A. cerana* venoms were similar to their melittins. Interestingly, an equal concentration of honey bee venoms and their melittins showed similarities in antimicrobial efficiency. Their pure melittins should have higher activity than the crude honey bee venoms.

Moreover, honey bee venoms extracted from *A. cerana* and *A. mellifera* were used at lower concentrations than their melittins against some Gram-negative bacteria and yeast. All the extracted venoms from four honey species presented higher antimicrobial activities than their melittins against *B. subtilis*. These results indicated that bee venoms extracted from different honey bee species vary in their compositions. The amount and composition of the lipid, protein, carbohydrate, and alkaline phosphatase were usually highest in *A. cerana*, followed by *A. mellifera* and *A. florea*. However, the amount of melittin in the venoms did not reflect the antimicrobial activity, as the melittin contained in *A. dorsata*, *A. mellifera*, *A. florea*, and *A. cerana* were 95.8 ± 3.2%, 76.5 ± 1.9%, 66.3 ± 8.6%, and 56.8 ± 1.8%, respectively [8], and these values were not related to the antimicrobial properties. Another reason is that crude venom contains other antimicrobial substances, mainly minor components that could synergize with melittin and increase the antimicrobial activity. Phospholipase A2 (PLA2) is one such antimicrobial substance in the venom. sPLA2-IIA has been reported to display activity against Gram-positive and Gram-negative bacteria [32] and *B. antiracist,* which produced a capsule [33].

Furthermore, it killed both methicillin-resistant staphylococci and vancomycin-resistant enterococci [34]. Additionally, the variability in bee venom composition is related to the honey bee species, stage, geographical origin, and condition, which also affect the antimicrobial activity. The bee venoms and melittin peptides may be potential sources of antimicrobial agents against Gram-positive and antibiotic-resistant bacteria. However, the antimicrobial activity derived from in vitro systems cannot cover all the biological variables found within the human body. The factors affecting the reliability of in vitro testing systems include the limitations in interpreting MIC, MBC, or MFC data due to the in vitro test conditions that cannot counterfeit the host environment and the variability of the testing media. Hence, in vivo studies of the kinetic, toxicity, and molecular mechanisms are still needed before further applications can be developed.

## 4. Materials and Methods

### 4.1. Collection and Preparation of the Bee Venom Extracts

Honey bee colonies were collected from 2016 to 2017, which included four honey bee species and three colonies of each species. Venom reservoirs were extracted by dissecting the stinging apparatus, and they were disrupted under light pressure on a glass slide using a sterile glass rod. The honey bee venom was washed in sterile deionized water, centrifuged at 10,000× *g* at 4 °C for 5 min to remove insoluble materials, and the supernatant was collected [15]. All samples were lyophilized and dissolved by phosphate-buffered saline (PBS), pH 7.4. The crude venom solution (1 mg/mL) of each honey bee species was filtered using a 0.22-µm syringe filter and kept at −20 °C before further experiments.

### 4.2. Genomic DNA Isolation

Total genomic DNA was isolated from four honey bee species using the ISOLATE II Genomic DNA Kit (Bioline, London, UK) in accordance with the manufacturer’s protocols. The DNA was stored at −80 °C until required.

### 4.3. Melittin Sequences

The extracted genomic DNA was used as a template for amplified melittin sequencing. This study designed the primers using Geneious version 8.1.8 (forward primer: 5′ GGA ATT CCA TAT GGG AAT TGG AGC AGT TC3′ and reverse primer: 5′GGC GCG GAT CCT TAT TAC TGT TGC CT3′). The melittin gene was synthesized using the KOD Hot Start Master Mix (Merck Millipore, Darmstadt, Germany). The thermal cycling conditions were as follows: one cycle of initial denaturation at 95 °C for 2 min, 35 cycles of denaturation at 95 °C for 20 s, annealing at 55 °C for 10 s, and extension at 70 °C for 10 s. The PCR products were purified using the PureLink^®^ Gel Extraction Kit (Life Technologies, Carlsbad, CA, USA) and sequencing (Macrogen Inc., Seoul, Korea).

### 4.4. Peptide Synthesis

Four sequences of the melittin peptide were chemically synthesized by SynPeptide Co., Ltd. (Shanghai, China) based on the deduced amino acid sequences, as shown in Table 1. A melittin working stock solution was prepared by dissolution in phosphate-buffered saline (pH 7.7) at a concentration of 1 mg/mL for the antimicrobial and antioxidant assays.

### 4.5. Tested Microorganisms

The antimicrobial properties of the crude venom from four honey bee species and melittin peptides were tested against five Gram-positive bacteria, including *Staphylococcus aureus* TISTR 517, *Staphylococcus epidermidis* DMST 15505, methicillin-resistant *Staphylococcus aureus* (*MRSA*) DMST 20625, *Bacillus subtilis* DMST 15896, and *Micrococcus luteus* DMST 15503, as well as three Gram-negative bacteria, including *Klebsiella pneumoniae* DMST 8216, *Salmonella typhimurium* DMST 562, and *Escherichia coli* ATCC 25922, and *Candida albicans* TISTR 5554 (yeast). All microorganisms were purchased from the American Type Culture Collection (ATCC), the Department of Medical Sciences Thailand (DMST), and the Thailand Institute of Scientific and Technological Research (TISTR).

### 4.6. Detection of Minimum Inhibitory and Minimum Bactericidal Concentrations (MIC and MBC)

All microorganisms that were used for the determination of the minimum inhibitory concentrations (MICs) were freshly prepared before testing. Each culture was transferred to a sterile PBS, and the turbidity was adjusted by the 0.5 McFarland Standard (Himedia, Mumbai, India). Each microorganism was added to the crude venom and melittin peptides at different concentrations (3.12–400 µg/mL) in 96-well plates following the Clinical and Laboratory Standards Institute (CLSI) guidelines [35,36]. Sterile PBS saline was used as a negative control, while 1 mg/mL of gentamicin (Merck KGaA, Darmstadt, Germany) and nystatin (Bio Basic Canada Inc., Markham, ON, Canada) was used as a positive control for the bacteria and yeast, respectively. All bacteria were incubated at 37 °C for 24 h, and the yeast was cultured at 30 °C for 48 h. The inhibition of microbial growth was determined by measuring the absorbance at 600 nm. The lowest concentration of venom or melittin peptide that inhibited microbial growth was the MIC. To determine the MBC/MFC, 0.1 mL of MIC with no microbial growth was spread onto Mueller–Hinton agar (HiMedia, Mumbai, India) at 37 °C for 24 h (for bacteria) or Sabouraud’s dextrose agar (HiMedia, Mumbai, India) at 30 °C for 48 h (for yeast). The final concentration of venom or melittin peptide that did not appear to contain microbial growth was the MBC/MFC.

### 4.7. Bioinformatics Analysis for Melittin Structures

The melittin gene sequences from *A. dorsata* and *A. florea* were obtained through this study, while the *A. mellifera* and *A. cerana* melittin sequences were retrieved from the National Center for Biotechnology Information (NCBI: https://www.ncbi.nlm.nih.gov/) (accessed on 10 March 2016) (Table 1). The deduction of the amino acid sequences of melittin and protein alignment were performed by using Geneious version 10.2.3. The secondary structures of all the melittins were predicted using SOPMA [37]. The amino acid compositions and physicochemical parameters, such as the molecular weights, predicted pI, GRAVY, instability index, and hydropathicity, were estimated using ProtParam [38] (http://web.expasy.org/protparam/) (accessed on 26 April 2021). For the analysis of the net charge, hydrophobic moment (mH), hydrophobicity (H), and melittin helical wheel plot, we used the online program HeliQuest [13] (http://heliquest.ipmc.cnrs.fr/cgi-bin/ComputParamsV2.py) (accessed on 26 April 2021).

### 4.8. Statistical Analysis

All the data analyses were performed using IBM SPSS Statistics Version 25.0 (IBM Corp., Armonk, NY, USA) and checked for normality of distribution. The Freidman and Wilcoxon signed-rank tests with Bonferroni adjustment for multiple comparisons were used to test the MIC and MBC/MFC data of the crude venoms against pathogenic microbes and test the MIC and MBC/MFC data of melittin peptides against pathogenic microbes as well. Comparisons of the MIC and MBC data among the three treatments in each honey bee species (crude venom, melittin peptide, and antibiotic) were made using the Kruskal–Wallis test followed by the Dunn–Bonferroni multiple comparison test. The level of significance was concluded to be *p* < 0.05.

## 5. Conclusions

To summarize, we successfully investigated melittin genes from four species of honey bees. Most of the melittins were similar in terms of size and the number of amino acids. Melittin and crude venom from *A. cerana* displayed the most antimicrobial activity against Gram-positive bacteria and *MRSA* compared to the other honey bees. Nevertheless, the antimicrobial activity of honey bee venoms and melittins against Gram-negative bacteria and fungi is limited. Slight differences in the amino acid sequence, size, structuring, charge, amphipathicity, and hydrophobicity may play an essential role in the melittin properties. The present investigation revealed that bee venoms and melittin peptides, the primary component of bee venom, also act as an antimicrobial peptide in bee venom that could be a promising candidate for use as an antimicrobial agent.

## Figures and Tables

**Table 1:** Melittin peptide sequences of four honey bee species.

No.	Species	Accession No.	Amino Acid Sequences	Reference
1	*A. mellifera*	AFI40556	GIGAVLKVLTTGLPALISWIKRKRQQG	[12]
2	*A. cerana*	P0DPR9.1	GIGAVLKVLTTGLPALINWIKRKRQQG	
3	*A. florea*	AMP82000	GIGAILKVLATGLPTLISWIKNKRKQG	This study
4	*A. dorsata*	AMP81999	GIGAILKVLSTGLPALISWIKRKRQEG	

**Table 2:** Amino acid composition (%) and physicochemical parameters of melittin from four honey bee species computed by ExPAsy ProtParam and HeliQuest. Shading indicates the four most abundant amino acids in each melittin.

	AMM	ACM	AFM	ADM
No. of AA	27	27	27	27
Mw (Da)	2904.54	2931.56	2876.52	2905.52
pI	12.02	12.02	11.33	11.10
GRAVY	0.248	0.148	0.281	0.256
Instability index (II)	43.44	39.60	34.64	50.58
Aliphatic index	130	130	133.7	133.7
Extinction coefficients	5500	5500	5500	5500
R−	0	0	0	1
R+	5	5	5	5
Hydrophobicity <H>	0.492	0.471	0.500	0.487
Hydrophobic moment <μH>	0.380	0.400	0.412	0.401
Net Charge (z)	5	5	5	4
Ala	7.4	7.4	7.4	7.4
Arg	7.4	7.4	3.7	7.4
Asn	0	3.7	3.7	0
Asp	0	0	0	0
Cys	0	0	0	0
Gln	7.4	7.4	3.7	3.7
Glu	0	0	0	3.7
Gly	14.8	14.8	14.8	14.8
His	0	0	0	0
Ile	11.1	11.1	14.8	14.8
Leu	14.8	14.8	14.8	14.8
Lys	11.1	11.1	14.8	11.1
Met	0	0	0	0
Phe	0	0	0	0
Pro	3.7	3.7	3.7	3.7
Ser	3.7	0	3.7	7.3
Thr	7.4	7.4	7.4	3.7
Trp	3.7	3.7	3.7	3.7
Tyr	0	0	0	0
Val	7.4	7.4	3.7	3.7
Pyl	0	0	0	0
Sec	0	0	0	0

AA: Amino acid, M_W_: the molecular weights, pI: isoelectric point, GRAVY: grand average of hydropathy, R−: the number of negative residues, and R+: the number of positive residues.

**Table 3:** Representation of the melittin secondary structure from honey bees predicted via SOPMA (in percentages).

	α-Helix	β-Turn	Random Coil	Extended Strand
AMM	40.74	33.33	18.52	7.41
ACM	59.26	22.22	18.52	0
AFM	51.85	22.22	14.82	11.11
ADM	55.55	29.63	7.41	7.41

**Table 4:** Minimum inhibitory concentrations (MICs) and minimum bactericidal concentrations (MBCs) of crude honey bee venom from four honey bee species against pathogenic bacteria.

Microorganism	MIC (μg/mL)				MBC/MFC (μg/mL)			
	*A. dorsata* ^c^	*A. mellifera* ^a^	*A. cerana* ^a^	*A. florea* ^b^	*A. dorsata* ^c^	*A. mellifera* ^a^	*A. cerana* ^a^	*A. florea* ^b^
**Gram-negative bacteria**								
*E. coli*	>400	266.7 ± 115.5	200.0 ± 0.0	>400	>400	400.0 ± 0.0	400.0 ± 0.0	>400
*K. pneumonia*	>400	400.0 ± 0.0	400.0 ± 0.0	>400	>400	400.0 ± 0.0	400.0 ± 0.0	>400
*S. typhimurium*	>400	>400	>400	>400	>400	>400	>400	>400
**Gram-positive bacteria**								
*B. subtilis*	133.3 ± 57.7	41.7 ± 14.4	37.5 ± 21.7	83.3 ± 28.9	133.3 ± 57.7	41.7 ± 14.4	37.5 ± 21.7	83.3 ± 28.9
*M. luteus*	66.7.0 ± 28.9	20.8 ± 7.2	16.7 ± 7.2	41.7 ± 14.4	66.7 ± 28.9	25.0 ± 0.0	25.0 ± 0.0	50.0 ± 14.4
*S. aureus*	266.7 ± 115.5	41.7 ± 14.4	41.7 ± 14.4	83.3 ± 28.9	266.7 ± 115.5	41.7 ± 14.4	41.7 ± 14.4	83.3 ± 28.9
*MRSA*	>400	66.7 ± 28.9	50.0 ± 0.0	200.0 ± 0.0	>400	100.0 ± 0.0	66.7 ± 28.9	400.0 ± 0.0
*S. epidermidis*	66.7.0 ± 28.9	16.7 ± 7.2	16.7 ± 7.2	33.3 ± 14.4	66.7 ± 28.9	16.7 ± 7.2	16.7 ± 7.2	50.0 ± 0.0
**Fungus**								
*C. albicans*	>400	333.3 ± 115.5	266.7 ± 115.5	>400	>400	333.3 ± 115.5	266.7 ± 115.5	>400

MIC: minimum inhibitory concentration, MBC: minimum bactericidal concentration, and MFC: minimum fungicidal concentration. MIC means were compared using the Friedman test (*p* < 0.001) and Wilcoxon signed-rank test with Bonferroni adjustment: *A. dorsata* and *A. mellifera* (*p* < 0.001), *A. dorsata* and *A. cerana* (*p* < 0.001), *A. dorsata* and *A. florea* (*p* = 0.013), *A. mellifera* and *A. cerana* (*p* = 0.395), *A. mellifera* and *A. florea* (*p* < 0.001), and *A. cerana* and *A. florea* (*p* < 0.001). The MBC/MFC means were compared using the Friedman test (*p* < 0.001) and Wilcoxon signed-rank test with Bonferroni adjustment: *A. dorsata* and *A. mellifera* (*p* < 0.001), *A. dorsata* and *A. cerana* (*p* < 0.001), *A. dorsata* and *A. florea* (*p* = 0.028), *A. mellifera* and *A. cerana* (*p* = 0.066), *A. mellifera* and *A. florea* (*p* < 0.001), and *A. cerana* and *A. florea* (*p* < 0.001). ^a^ > ^b^ > ^c^ in potency.

**Table 5:** Minimum inhibitory concentrations (MICs) and minimum bactericidal concentrations (MBCs) of melittin from four honey bee species against pathogenic bacteria.

Microorganism	MIC (μg/mL)				MBC/MFC (μg/mL)			
	ADM ^c^	AMM ^b^	ACM ^a^	AFM ^b,c^	ADM ^c^	AMM ^a,b^	ACM ^a^	AFM ^b^
**Gram-negative bacteria**								
*E. coli*	>400	>400	400 ± 0.0	>400	>400	>400	>400	>400
*K. pneumonia*	>400	>400	>400	>400	>400	>400	>400	>400
*S. typhimurium*	>400	>400	>400	>400	>400	>400	>400	>400
**Gram-positive bacteria**								
*B. subtilis*	>400	>400	400 ± 0.0	>400	>400	>400	400 ± 0.0	>400
*M. luteus*	50 ± 0.0	25 ± 0.0	25 ± 0.0	25 ± 0.0	50 ± 0.0	25 ± 0.0	25 ± 0.0	25 ± 0.0
*S. aureus*	200 ± 0.0	50 ± 0.0	50 ± 0.0	50 ± 0.0	200 ± 0.0	50 ± 0.0	50 ± 0.0	50 ± 0.0
*MRSA*	400 ± 0.0	100 ± 0.0	100 ± 0.0	200 ± 0.0	>400	100 ± 0.0	100 ± 0.0	200 ± 0.0
*S. epidermidis*	50 ± 0.0	25 ± 0.0	12.5 ± 0.0	50 ± 0.0	50 ± 0.0	25 ± 0.0	12.5 ± 0.0	50 ± 0.0
**Fungus**								
*C. albicans*	>400	>400	>400	>400	>400	>400	>400	>400

MIC: minimum inhibitory concentration, MBC: minimum bactericidal concentration, and MFC: minimum fungicidal concentration. MIC means were compared using the Friedman test (*p* < 0.001) and Wilcoxon signed-rank test with Bonferroni adjustment: ADM and AMM (*p* = 0.011), ADM and ACM (*p* = 0.001), ADM and AFM (*p* = 0.107), AMM and ACM (*p* = 0.034), AMM and AFM (*p* = 0.154), and ACM and AFM (*p* = 0.011). The MBC/MFC means were compared using the Friedman test (*p* < 0.001) and Wilcoxon signed-rank test with Bonferroni adjustment: ADM and AMM (*p* = 0.011), ADM and ACM (*p* = 0.004), ADM and AFM (*p* = 0.042), AMM and ACM (*p* = 0.146), AMM and AFM (*p* = 0.146), and ACM and AFM (*p* = 0.042). ^a^ > ^b^ > ^c^ in potency.

## Data Availability

The data presented in this study are available upon request from the corresponding author.
